# Functional single-cell omics in gynecological cancers immunotherapy: from heterogeneity dissection to clinical translation

**DOI:** 10.3389/fimmu.2026.1981010

**Published:** 2026-10-12

**Authors:** Dong-mei Li, Kai-ge Pei, Bo-wen Yang, Ming-rong Qie, Jin-feng Xu, Lan Zhong, Liang Song

**Affiliations:** 1 Department of Obstetrics and Gynecology, West China Second University Hospital, Sichuan University, Chengdu, China; 2 Key Laboratory of Birth Defects and Related Diseases of Women and Children (Sichuan University), Ministry of Education, Chengdu, China; 3 Department of Medical Oncology, West China Second University Hospital, Sichuan University, Chengdu, China

**Keywords:** gynecological cancer, immunotherapy, single-cell omics, T cells, TILs

## Abstract

Ovarian, endometrial, and cervical cancers represent major threats to women’s health worldwide. The profound heterogeneity of their immune microenvironments has rendered immune checkpoint blockade (ICB) therapies, particularly PD-1/PD-L1 inhibitors, far less effective than anticipated in gynecological malignancies. Conventional research approaches, constrained by coarse analytical resolution, are insufficient to unravel the underlying mechanisms of this therapeutic shortfall. Functional single-cell omics technologies, including single-cell RNA sequencing, single-cell chromatin accessibility profiling, spatial transcriptomics, and multi-omics integration, have provided a new framework for systematically studying the tumor immune microenvironment across three progressive dimensions: cellular identity, functional state, and spatial localization. This review systematically synthesizes the applications of this technological toolkit in the three major gynecological cancers. It reveals distinct bottlenecks across the three dimensions. In ovarian cancer, the core obstacle lies in the spatial dimension, where cancer-associated fibroblast-mediated physical barriers and pro-angiogenic macrophage subsets together construct a “spatial exclusion” microenvironment. In cervical cancer, the principal bottleneck resides in the functional dimension, where HPV status and histological subtype drive a “functional disability” phenotype, in which T cells infiltrate but fail to achieve effective activation, with therapy resistance-associated immune evasion clones further exacerbating functional impairment. In endometrial cancer, however, the immune fate is governed by the identity dimension, where molecular subtypes dictate the baseline nature of immune infiltration, and autophagy-related cellular subpopulations may offer new insights into intratumoral heterogeneity. Although single-cell omics has identified immunologically actionable targets with translational potential, including TREM2^+^ macrophages and the LAG-3/TIM-3 dual-target axis, clinical translation remains constrained by technical and biological bottlenecks, including stringent requirements for tissue sample quality, high assay costs, lack of standardized analytical pipelines, and the discordance between transcriptomic profiles and protein-level functionality.

## 1 Introduction

Ovarian, endometrial, and cervical cancers are the three major gynecological malignancies and account for a substantial portion of the global cancer burden among women. Cervical cancer ranks as the fourth most common malignancy in women worldwide, with over 600,000 new cases diagnosed annually (1). Although ovarian cancer has a lower incidence rate than cervical cancer, it remains the most lethal gynecological malignancy due to its insidious onset and profound heterogeneity, accounting for approximately 210,000 female deaths each year. Tumor heterogeneity profoundly influences growth kinetics, invasive potential, and drug sensitivity, thereby critically shaping patient prognosis (2, 3). Meanwhile, the incidence of endometrial cancer has been steadily rising in parallel with the global epidemic of metabolic syndrome (4).

Despite the substantial clinical benefits achieved with immune checkpoint blockade (ICB) therapies, particularly PD-1/PD-L1 inhibitors, in multiple solid tumor types, their efficacy in gynecological malignancies has fallen considerably short of expectations. In high-grade serous ovarian cancer (HGSOC) in particular, the objective response rate to PD-1/PD-L1 monotherapy is less than 20% (5–7). This raises a fundamental question: why does the immune system exhibit divergent response states across different gynecological cancers, despite their shared classification as “malignancies”? The three major gynecological cancers display strikingly distinct immune response patterns. Ovarian cancer is characterized predominantly by a “spatial exclusion” phenotype, wherein tumor-infiltrating lymphocytes (TILs) either fail to penetrate the tumor parenchyma or remain confined to the stroma and peripheral margins (6, 8), suggesting the presence of physical or chemotactic barriers within the tumor microenvironment that impede effective spatial access of effector immune cells. Cervical cancer, driven by persistent high-risk HPV infection, is abundant in TILs; however, immune functionality is constrained by pronounced enrichment of immunosuppressive signals, notably transforming growth factor-beta (TGF-β), resulting in a “functional disability” phenotype—T cells are present but “hot yet inert”, with their effector functions restrained by the microenvironmental signaling network (9). Endometrial cancer, in contrast, exhibits a typical “subtype-driven” phenotype, wherein immune features are largely dictated by molecular subtypes: the DNA polymerase epsilon (POLE) ultramutated subtype is distinguished by high tumor mutational burden and dense immune infiltration, whereas the p53-mutated subtype presents as a near-immune desert, suggesting that molecular classification fundamentally determines the immune fate and, consequently, the response to immunotherapy (10).

The therapeutic predicament described above does not arise because of the fundamental cellular nature of these tumors. It does not arise because tumors have been misclassified as ‘hot’, ‘cold’, or ‘immune-desert’. Rather, it arises because conventional bulk analytical approaches lack the resolution to dissect the mechanistic heterogeneity that operates within a given immune phenotype. The advent of single-cell technologies has not challenged the classical immune phenotype framework; instead, it has substantially enriched our understanding of the cellular origins, functional states, and spatial constraints that produce these phenotypes. In other words, single-cell omics does not redefine whether a tumor is hot or cold; it explains why a tumor that appears ‘hot’ by conventional criteria may nonetheless fail to respond to immunotherapy. This review synthesizes the research progress in translating functional single-cell omics discoveries into precision immunotherapy for gynecological malignancies, and examines, from the three dimensions of cellular identity, functional state, and spatial localization, the major challenges that tumor heterogeneity poses for clinical translation.

## 2 Three-dimensional analytical framework of functional single-cell omics and cellular heterogeneity

In this review, we introduce the term “functional single-cell omics” to describe an integrative analytical framework that goes beyond cellular identity to also capture functional state and spatial context, that is, not only what a cell is, but also what it is doing, where it resides, and which cells it interacts with. This framework differs from conventional ‘single-cell omics’, which is primarily identity-focused, and from ‘single-cell multi-omics’, which refers to the simultaneous measurement of multiple molecular modalities from the same cell. Conventional bulk RNA sequencing (bulk RNA-seq) captures averaged transcriptional signals from tissue samples, wherein the heterogeneity across distinct cell types, functional states, and spatial localizations is masked within population-level means, thereby precluding the resolution of subtle but critical differences among cellular subpopulations (11). Single-cell RNA sequencing (scRNA-seq) has overcome this limitation. Since Tang et al. (12) first achieved whole-transcriptome (mRNA) sequencing of individual mouse blastomere cells in 2009, scRNA-seq has undergone rapid development, ushering molecular biology research into a new era of cellular-resolution profiling. By enabling independent transcript capture and sequencing of individual cells, scRNA-seq allows precise delineation of the identity characteristics, functional states, and molecular phenotypes of each cell (13, 14).

Functional single-cell omics constitutes a continuously evolving methodological framework, the core inquiry of which is to determine the precise functional role of each individual cell within the tumor microenvironment. This interrogation must be advanced progressively across three dimensions: identity, state, and space. Identity confirmation relies primarily on scRNA-seq, which enables unbiased clustering based on whole-genome transcriptional profiles to discriminate cellular lineage origins (e.g., T cells, macrophages, dendritic cells, or fibroblasts) (15, 16). However, transcriptome-based identity assignment ultimately represents a “static label” that fails to elucidate the functional propensity of a given cell within its current microenvironmental context. To address this, state assessment employs single-cell chromatin accessibility sequencing (scATAC-seq) to reveal alterations in chromatin openness at the epigenetic regulatory level (17, 18), or utilizes functional gene set scoring to quantitatively dimension the functional attributes of single-cell transcriptomic data, thereby determining whether a cell resides in an activated, exhausted, quiescent, pro-inflammatory, or anti-inflammatory functional state (19, 20). Nevertheless, both scRNA-seq and scATAC-seq rely on information derived from dissociated single-cell suspensions following tissue digestion, a process during which the spatial localization information of cells within their native tissue architecture is entirely lost (21).

This limitation has catalyzed a technological breakthrough in the spatial localization dimension. Spatial transcriptomics has emerged as a complementary approach, enabling high-throughput gene expression profiling while preserving the spatial architecture of tissues, thereby offering novel perspectives for dissecting cellular spatial organization and biological processes. This technology has become a focal point in the life sciences and was named the Method of the Year 2020 by Nature Methods (22). Spatial transcriptomics assigns spatial coordinates to single-cell information, concurrently delineating cell localization and neighboring intercellular interactions (23–25). The transition from population-averaged measurements to single-cell resolution, further augmented by the spatial dimension, represents a technological leap that constitutes the cornerstone of single-cell omics-driven advances in tumor immune microenvironment research (23, 26).

It is noteworthy that the three dimensions of identity, state, and space do not constitute parallel and independent streams of information, but rather form a cognitive continuum with inherent logical progression (27, 28). Identity confirmation delineates the framework of cell types; state assessment transforms static labels into dynamic functional descriptions; and spatial localization integrates molecular information with tissue architecture. These three dimensions are progressively nested, culminating in a tripartite “identity–state–space” analytical framework (29, 30). Only through systematic integration of information across all three dimensions can the interpretation of the tumor immune microenvironment transcend the conventional static inventory of “which cell types are more or less abundant”. It can then truly rise to a multidimensional landscape that integrates molecular resolution, functional dynamics, and spatial architecture, thereby providing a more precise coordinate system for subsequent mechanistic exploration and targeted intervention. In the following sections, we apply this framework to re-examine the distinct immune microenvironment profiles of the three major gynecological cancers: spatial exclusion in ovarian cancer, functional disability in cervical cancer, and subtype-driven features in endometrial cancer. We further elucidate how single-cell omics addresses each of these clinical challenges through its corresponding analytical dimension.

## 3 Four core single-cell technologies and gynecological cancers

Single-cell sequencing technologies have evolved into powerful tools for dissecting intercellular interactions and resolving the heterogeneity of the tumor microenvironment, offering novel perspectives for clinical tumor management. The four major technological systems, namely scRNA-seq, scATAC-seq, spatial transcriptomics, and multi-omics integration, progressively dissect the cellular composition and functional networks of the tumor immune microenvironment at the levels of transcriptional profiling, epigenetic regulation, spatial localization, and multidimensional information integration, respectively (20, 31, 32). Their functional positioning and integrated application are summarized in **Figure 1**. In 2017, scRNA-seq was first applied to the study of immune cells in ovarian cancer ascites, marking the beginning of the single-cell research era in gynecological oncology (33). This approach was later extended by Izar et al., who profiled ~11,000 cells from 22 ascites specimens and provided a comprehensive single-cell landscape of the HGSOC ascites ecosystem (34).

**Figure 1:**
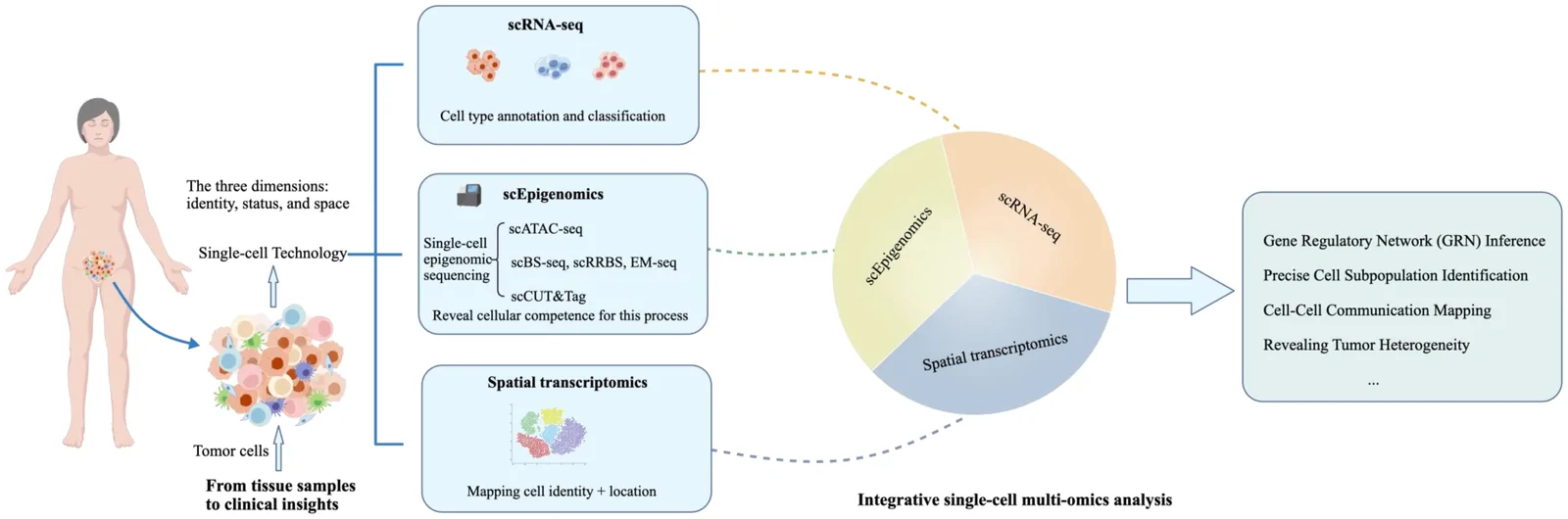
Functional positioning and integrated application of single-cell and spatial multi-omics technologies. Single-cell RNA sequencing (scRNA-seq) is primarily employed for cell type annotation and classification. Single-cell epigenomic sequencing (scATAC-seq, scBS-seq, scRRBS, EM-seq) and scCUT&Tag are utilized for gene regulatory network (GRN) inference and assessment of cellular functional competence, respectively. Spatial transcriptomics integrates cellular identity with spatial location information, enabling the construction of cell–cell communication networks, identification of fine-scale subpopulations, and spatial dissection of tumor heterogeneity. Each individual omics modality offers a restricted view of biological complexity, and thus cannot comprehensively recapitulate intricate biological processes. In contrast, integrative analysis of multi-omics data enables the establishment of an “identity–state–space” tripartite analytical framework within the native tissue context, thereby facilitating systematic elucidation of the molecular mechanisms and spatial architecture underlying tumor heterogeneity.

### 3.1 Single-cell RNA sequencing

Within the technological matrix of functional single-cell omics, scRNA-seq is one of the most widely applied technologies. This technique involves the capture, amplification, and sequencing of the transcriptome of individual cells, enabling the clustering of cells based on the similarity of gene expression profiles, and the inference of cellular differentiation trajectories through methods such as pseudo-time analysis (14). In gynecological cancer research, the core value of scRNA-seq lies in its capacity to uncover novel immune cell subsets that have been masked by bulk sequencing. For instance, in ovarian cancer research, investigators have successfully identified a macrophage subpopulation with pro-angiogenic functions (Angio-Mac) using this technology, and confirmed that its function is closely associated with aberrant activation of the NF-κB signaling pathway (35). This finding provides single-cell resolution molecular evidence for dissecting tumor heterogeneity from the cellular identity dimension. Beyond ovarian cancer, scRNA-seq has been extensively applied to cervical and endometrial cancers. In cervical cancer, scRNA-seq has been used to characterize the immune landscape of HPV-positive versus HPV-negative tumors, revealing that HPV-positive samples are enriched in CD4^+^ T cells and cDC2s, whereas HPV-negative tumors are predominantly infiltrated by CD8^+^ T cells (36). In endometrial cancer, scRNA-seq has resolved the immune microenvironment of TCGA molecular subtypes at high resolution, demonstrating that the POLE ultramutated subtype exhibits robust CD8^+^ effector T cell activation, whereas the NSMP subtype frequently presents an immune desert phenotype (37–40).

### 3.2 Single-cell epigenomic sequencing

If scRNA-seq addresses the question of “what a cell is and what it might do”, then scATAC-seq seeks to answer “why a cell does what it does” (41). This technique identifies active regulatory elements by detecting chromatin openness across the genome, thereby revealing the underlying logic of gene expression regulation at the epigenetic level. The work of Riegel et al. systematically demonstrated the unique value of single-cell epigenomic sequencing in tumor immunology research. Using scATAC-seq, they uncovered cell-state-specific chromatin accessibility heterogeneity that cannot be resolved by conventional bulk sequencing, and identified two distinct states of tumor-infiltrating CD8^+^ T cells, namely precursor exhausted T cells and terminally exhausted T cells, with significant differences in non-coding regulatory elements between them (42). Furthermore, scATAC-seq enables unbiased discovery of novel immune regulatory targets. In a single-cell multi-omics study of melanoma, scATAC-seq localized an open chromatin region upstream of the CD2 gene in T cells, which contains binding motifs for multiple transcription factors including CREB1 and JUN, providing direct physical evidence that CD2 expression is controlled by specific epigenetic regulatory elements (43). In other words, scATAC-seq reveals not the “phenomenon” of functional loss in T cells, but the “mechanism” by which function is epigenetically “locked”. The former represents transcriptional fluctuation, whereas the latter represents homeostatic remodeling at the chromatin level. It is precisely this capacity to dissociate and localize causality at the epigenetic dimension that establishes scATAC-seq as a key technological approach for dissecting the mechanisms governing cellular functional states within the tumor immune microenvironment.

### 3.3 Spatial transcriptomics

Although the aforementioned two technologies are capable of providing abundant molecular information, they share a common limitation, namely the loss of original spatial localization information of cells during tissue dissociation. Spatial transcriptomics (ST) emerged precisely to address this issue (44, 45). This technology employs capture spots with spatial barcodes on tissue sections, enabling the acquisition of transcriptomic data from each capture spot while preserving the spatial architecture of the tissue (46). In HGSOC research, spatial transcriptomics combined with single-cell sequencing has directly confirmed at the spatial level a long-speculated phenomenon: cancer-associated fibroblasts (CAFs), through TGF-β/SMAD signaling-driven remodeling of the extracellular matrix (ECM), construct a dense fibrotic barrier at the tumor margin, composed predominantly of collagen and fibronectin, which excludes CD8^+^ T cells from the tumor core, thereby constituting the structural basis of the “immune-excluded” microenvironment (6, 47, 48). This finding not only explains the spatial reasons for the poor response of ovarian cancer to immune checkpoint inhibitors, but also provides a theoretical rationale for targeting CAFs to relieve the spatial barrier, elucidating from the spatial localization dimension the microenvironmental causes of restricted T cell spatial accessibility. In cervical cancer, spatial transcriptomics has further demonstrated a strong spatial interaction between CD8^+^ T cells and epithelial subtypes in squamous cell carcinoma, which is markedly diminished in adenocarcinoma (49). In endometrial cancer, high-plex spatial transcriptomic profiling has revealed distinct immune components in mismatch repair-deficient tumors and highlighted a HLA class I/DNMT3A/CD8 modulatory axis that may shape CD8^+^ T cell infiltration (50).

### 3.4 Single-cell multi-omics integration

#### 3.4.1 Single-cell proteomic technologies (CyTOF)

A single technology often provides only “snapshot” information. For instance, although scRNA-seq can identify a novel macrophage subpopulation, it cannot determine whether it actually suppresses T cells (which requires protein-level validation), nor whether it is located in the tumor core (which requires spatial localization) (51–53). Single-cell proteomic technologies, represented by mass cytometry (CyTOF), extend the analytical dimension to the protein level (54, 55). This technology utilizes metal isotope-labeled antibodies to label surface or intracellular proteins of single cells, followed by quantitative detection via time-of-flight mass spectrometry, enabling simultaneous measurement of dozens of functional proteins at the single-cell level (54, 56). CyTOF enables simultaneous quantification of dozens of immune markers at the single-cell level using metal isotope-labeled antibodies, and has been increasingly applied to dissect the immune microenvironment in gynecological tumors (57).

#### 3.4.2 Multi-omics integration in gynecological cancers

Multi-omics integration can be broadly categorized into vertical integration, which combines multiple molecular modalities measured from the same cell (e.g., scRNA-seq plus scATAC-seq), and horizontal integration, which aligns data from different platforms or samples (e.g., scRNA-seq plus spatial transcriptomics). Commonly used computational strategies include early integration, in which data matrices are concatenated prior to dimensionality reduction; intermediate integration, in which shared latent spaces are learned across modalities (e.g., Seurat, Harmony, MOFA); and late integration, in which modality-specific predictions are combined at the downstream analytical level. A comprehensive study of HGSOC integrating scRNA-seq and spatial transcriptomics revealed significant differences in the immune microenvironment between primary tumors and omental metastases: the primary site exhibited a more active immune status, whereas metastatic lesions were enriched with immunosuppressive signals, suggesting spatial and temporal heterogeneity of immune states across different tumor regions (58). Beyond transcriptome-spatial integration, matched scRNA-seq and scATAC-seq profiling of ovarian and endometrial tumors has linked chromatin accessibility variation to gene expression, revealing previously unannotated regulatory elements that drive hallmark cancer pathways and highlighting the importance of intratumoral heterogeneity (59). In cervical cancer, multi-omics profiling combining proteomics and metabolomics has uncovered sphingolipid metabolism reprogramming in myeloid-derived suppressor cells, identifying the Kng1-sphingosine 1-phosphate axis as a central protein-metabolite regulatory node (60). The aforementioned cross-technology, cross-dimensional integrative analysis synergistically addresses the core demands of dissecting immune microenvironment heterogeneity in gynecological malignancies from the three dimensions of cellular identity, functional state, and spatial localization. Collectively, multi-technology integration has become an increasingly important strategy in current research, although many single-cell studies still rely on a single modality due to cost, sample availability, and technical constraints.

## 4 Single-cell characteristics of the immune microenvironment in three major gynecological cancers

### 4.1 Ovarian cancer: the “cold immune” dilemma under high heterogeneity

#### 4.1.1 Clinical background and the necessity of single-cell research

A clinical genomics study in Japan demonstrated that among the most common HGSOC subtype, over 90% harbor TP53 mutations, which are associated with poor prognosis (61). The molecular heterogeneity of HGSOC primarily stems from impaired DNA repair, particularly homologous recombination deficiency (HRD), with approximately half of HGSOC cases being HRD-positive, most commonly attributable to BRCA mutations (62–65). Although BRCA-mutant tumors exhibit higher TIL infiltration due to a higher neoantigen burden, their CD8^+^ T cells are more prone to exhaustion and show diminished responsiveness to PD-1/PD-L1 inhibitor reinvigoration (66, 67). Why do tumors that are “T cell-rich” still fail to respond? Single-cell technologies offer a novel explanation.

Previous studies have broadly classified TAMs into M1 and M2 subtypes; however, this binary framework is insufficient to encompass the functional spectrum of TAMs within the actual tumor microenvironment (68). A large-scale integrative study, encompassing 111 samples with 272,389 CD45^+^ immune cells, constructed a single-cell atlas of the immune microenvironment across these three major gynecological cancers for the first time, identifying 11 macrophage subpopulations, among which two extremes were particularly critical: NFKB1-driven Angio-Mac (characterized by high expression of VCAN/THBS1/VEGFA, activating NF-κB to promote angiogenesis and immunosuppression, and associated with poor prognosis), and PITX1-regulated IFN-Mac_CXCL9 (secreting CXCL9/10/11 to recruit T cells via the CXCR3 axis, and correlated with improved survival) (35). This provides mechanistic clues to explain the clinical paradox of “abundant T cell infiltration but limited immunotherapy response” in BRCA-mutant tumors: even if T cells are physically present in the tumor, their function may be constrained by the immunosuppressive microenvironment driven by TAMs (such as the Angio-Mac subpopulation). Related studies have also confirmed the enrichment of an immunosuppressive network dominated by Trem2/ApoE-high TAMs in recurrent tumors (69). Collectively, these findings argue against indiscriminate TAM depletion and instead prioritize the selective elimination of pro-tumorigenic subsets.

#### 4.1.2 Molecular subtypes of ovarian cancer and single-cell multi-omics insights into clinical outcomes

Epithelial ovarian cancer (EOC) comprises distinct histological subtypes with unique molecular features and clinical behaviors: HGSOC accounts for approximately 75%, whereas low-grade serous (LGSOC), clear cell (OCCC), endometrioid (ENOC), and mucinous (MOC) carcinomas each account for approximately 5–10% (70). The genomic alteration landscape differs markedly across subtypes: HGSOC is characterized by TP53 mutations in 96% of tumors and homologous recombination repair gene (BRCA1/2) defects in approximately half of cases; OCCC frequently harbors ARID1A and PIK3CA mutations; ENOC is dominated by PIK3CA and KRAS alterations; and MOC and LGSOC are characterized by CDKN2A/KRAS and MAPK pathway gene (BRAF/KRAS) alterations, respectively (70–72). Prognosis differs significantly among these histological subtypes, with LGSOC showing the most favorable outcome and HGSOC and ovarian carcinosarcoma being the most aggressive (72–74). Within HGSOC, integrated multi-omics analyses have progressively refined molecular subtyping. The TCGA classified HGSOC into mesenchymal, immunoreactive, differentiated, and proliferative subtypes based on gene expression (75); the CPTAC subsequently performed comprehensive proteomic, phosphoproteomic, and glycoproteomic characterization of 174 ovarian tumors (169 HGSOC), revealing how copy-number alterations influence the proteome and identifying protein acetylations associated with homologous recombination deficiency, though without establishing prognostic proteomic subtypes (76). More recently, Tu et al. performed proteomic analysis of 116 primary HGSOC tumors and identified three reproducible proteomic subtypes with distinct clinical outcomes: S-I (immune-active), S-II (metabolic), and S-III (extracellular matrix dynamic), among which S-II exhibited the best prognosis and S-III the worst, with S-III significantly associated with advanced-stage disease and immune escape (77). In parallel, Tan et al. performed an integrated proteomic and scRNA-seq study of 154 EOC tumor samples and 29 normal fallopian tube tissues, together with scRNA-seq of 8 additional EOC tumors, and identified four proteomic subtypes with distinct clinical significance: malignant proliferative (C1), immune-infiltrating (C2), fallopian-tube-like (C3), and differentiated (C4) (78, 79). The C2 subtype was characterized by enrichment of GZMK^+^ CD8^+^ T cells and phagocytic MRC1^+^ macrophages. CD40 was identified as a subtype-specific prognostic factor for C2, with interactions between CD40^+^ phagocytic macrophages and CD40L^+^IL17R⁻CD4^+^ T cells significantly correlating with favorable prognosis. In patients with non-immune subtypes, TYMP inhibition was validated as a promising therapeutic strategy, with TAS102 (targeting TYMP) combined with 5-FU suggested as a potential regimen (78). Collectively, these findings illustrate how single-cell multi-omics can link molecular subtypes to cellular networks and clinical outcomes, providing a rational basis for subtype-guided therapeutic strategies.

#### 4.1.3 Cancer-associated fibroblast triple barriers and spatial exclusion

Cancer-associated fibroblasts (CAFs) are critical components of the tumor microenvironment (TME) and play pivotal roles in cancer progression and therapeutic response (79, 80). According to the tumor immune phenotype (TIPs) classification framework, the ovarian cancer microenvironment can be categorized into three functional states: immune-inflamed, immune-excluded, and immune-desert (81–85). Among these, the immune-excluded phenotype is the most typical, characterized by the presence of T cells in the stromal region but their inability to penetrate the tumor parenchyma. However, TIPs classification is based on morphological observations of tissue sections and cannot reveal the cellular origins or molecular composition of the exclusion barriers. Single-cell RNA sequencing has provided a refined analytical tool for this purpose. It should be noted that the iCAF/myCAF/apCAF nomenclature was initially established in pancreatic ductal adenocarcinoma and colorectal cancer (86), and direct validation of all three populations in ovarian cancer remains incomplete. In ovarian cancer specifically, myCAF-mediated collagen remodeling and spatial T cell exclusion have been directly demonstrated by spatial transcriptomics and single-cell analyses (87), whereas the immunomodulatory functions of apCAFs and the chemokine-driven MDSC recruitment by iCAFs are primarily extrapolated from other tumor types and have not been definitively established in ovarian cancer. With this caveat, the three subtypes are described as follows: inflammatory CAFs (iCAFs) secrete inflammatory chemokines such as IL-6 and CXCL1, recruiting myeloid-derived suppressor cells (MDSCs) and indirectly suppressing T cell responses (88); myofibroblastic CAFs (myCAFs) secrete dense collagen and remodel the extracellular matrix, forming physical barriers that spatially impede T cell infiltration (89, 90); and antigen-presenting CAFs (apCAFs), although expressing MHC-II molecules, lack co-stimulatory molecules such as CD80/CD86, potentially inducing Treg differentiation or T cell anergy (86, 91, 92). Among these three subtypes, myCAFs provide the structural barrier, while iCAFs and apCAFs synergistically amplify the exclusion effect through inflammatory chemotactic and immunoregulatory pathways. Spatial transcriptomics has further confirmed that in the immune-excluded microenvironment of ovarian cancer, myCAF-enriched regions are accompanied by significant collagen deposition and restricted T cell spatial localization (47, 93), directly validating from the spatial dimension the structural basis of CAF subpopulation-mediated immune exclusion.

#### 4.1.4 Immune differences between primary and metastatic sites

The unique intraperitoneal dissemination pattern of ovarian cancer results in significant differences in the immune microenvironment between primary tumors (ovary/fallopian tube) and metastatic lesions (omentum). Integrated analysis of single-cell and spatial transcriptomics revealed that primary lesions exhibit higher immune activity than omental metastases, and in untreated primary lesions, terminally differentiated CD4^+^ and CD8^+^ T cells predominate, with their dysfunction associated with altered expression of interferon alpha/gamma pathways in epithelial cells, suggesting that epithelial-immune crosstalk constitutes an important mechanism of immunosuppression in HGSOC (58). Further studies have demonstrated that neoadjuvant chemotherapy reshapes the microenvironmental ecology: post-chemotherapy, HLA diversity decreases, PDCD1/CD274 expression is upregulated in specific cell populations such as CD8_ANXA1 and LAMP3^+^ dendritic cells, while certain poor-prognosis-associated subpopulations, including cancer.cell.2 and CAF_C3, show enrichment trends in metastatic lesions and post-chemotherapy samples (94). Collectively, these findings indicate that the immune microenvironment of HGSOC is not only regulated by anatomical site (primary versus metastatic) (95), but is also dynamically shaped by therapeutic pressure, with both factors constituting important dimensions of tumor spatiotemporal heterogeneity.

### 4.2 HPV-driven immune microenvironment and resistance heterogeneity in cervical cancer

#### 4.2.1 HPV status reshapes the immune microenvironment

Fundamental differences exist in the immune microenvironment between HPV-positive and HPV-negative cervical cancers. Integrated single-cell and spatial transcriptomic analyses revealed that HPV-positive samples are enriched in CD4^+^ T cells and cDC2s, whereas HPV-negative tumors are predominantly infiltrated by CD8^+^ T cells. Mechanistically, in HPV-positive tumors, epithelial cells regulate cDC2s via the ANXA1-FPR1/3 axis, which in turn influences CD4^+^ T cells and interferon-related CD8^+^ T cell subsets; in HPV-negative tumors, epithelial cells primarily affect the monocyte-macrophage lineage, which subsequently interacts with CD8^+^ T cells (36). This finding reveals, from the cellular identity dimension, the fundamental remodeling effect of HPV status on immune cell composition, suggesting that immunotherapeutic strategies may require differential design according to distinct HPV statuses.

#### 4.2.2 Immune differences between histological subtypes

The two major histological subtypes of cervical cancer, squamous cell carcinoma (SCC) and adenocarcinoma (AC), exhibit distinct immune microenvironment profiles. Integrated single-cell and spatial transcriptomic analyses revealed that AC is enriched in epithelial cells and displays a “stroma-rich, immune-cold” phenotype, whereas SCC is more immunogenic and enriched in plasma cells and NK/T cells. Spatial transcriptomics further demonstrated a strong spatial interaction between CD8^+^ T cells and epithelial subtypes in SCC, which was markedly diminished in AC. The study also identified subtype-specific immune and stromal features, including ICOS^+^ Tregs, IDO1^+^, CAFs, and PLVAP^+^ endothelial cells, which may respectively drive immune evasion, angiogenesis, and metastasis (49). These findings reveal, from the spatial dimension, how histological subtypes shape differential microenvironments by altering the spatial distribution and interaction patterns of immune cells, providing a theoretical basis for combining immune checkpoint inhibitors with stromal-targeted agents.

#### 4.2.3 Three-dimensional dissection of the “functional disability” microenvironment in cervical cancer

Synthesizing the single-cell findings from the above three levels, the essence of the “functional disability” immune barrier in cervical cancer can be understood within a three-dimensional framework: HPV status dictates the identity basis of immune cell composition (HPV^+^ tumors enriched in CD4^+^ T cells and cDC2s, HPV⁻ tumors enriched in CD8^+^ T cells); histological subtypes shape the spatial landscape of immune-tumor interactions by modulating the intensity of spatial crosstalk between CD8^+^ T cells and epithelial subtypes; and CD47^+^ drug-resistant clones persisting after chemoradiotherapy represent an extreme phenotype of functional disability, wherein even when T cells are present within the microenvironment, their effector functions remain constrained by immune checkpoint signals. Together, these three dimensions elucidate the nature of “TILs present but inert” in cervical cancer, suggesting that clinical strategies should shift from sole immune checkpoint blockade toward combination regimens that relieve functional suppression, such as anti-TGF-β or anti-CD47 therapies.

### 4.3 Single-cell dissection of molecular subtypes and immune status in endometrial cancer

#### 4.3.1 Immunological essence of molecular subtypes

The TCGA molecular classification, encompassing POLE ultramutated, microsatellite instability (MSI), p53-abnormal (p53abn), and no specific molecular profile (NSMP) subtypes, has become an important stratifying basis in the clinical management of endometrial cancer (96). Single-cell RNA sequencing has resolved the immune microenvironment of each subtype at high resolution. The POLE ultramutated subtype exhibits an extremely high tumor mutational burden (TMB) and abundant neoantigens, with significant enrichment and robust activation of CD8^+^ effector T cells in the microenvironment, along with upregulated expression of immune checkpoint molecules such as PD-1/PD-L1, constituting the “hot tumor” phenotype most sensitive to immunotherapy (37–39). The p53abn subtype, predominantly represented by serous carcinoma, displays genomic instability; however, a large-cohort study by Martin et al. involving 256 cases revealed intratumoral immune heterogeneity within this subtype: over 50% of cases belong to TIL-enriched subpopulations, which are associated with improved overall survival and disease-specific survival, yet the overall microenvironment of this subtype remains predominantly immunosuppressive (10, 97, 98). The MSI subtype exhibits an intermediate level of immune infiltration between the POLE ultramutated and p53abn subtypes, characterized by the coexistence of CD8^+^ cytotoxic T lymphocytes and regulatory T cells (Tregs), with dual regulatory signals of both immune activation and immunosuppression (99). In contrast, the NSMP subtype, defined as “no specific molecular profile”, represents the most heterogeneous subgroup within the endometrial cancer molecular classification, accounting for approximately 35% to 45% of endometrioid carcinomas (100). Single-cell RNA sequencing has revealed that the NSMP subtype displays the most dispersed distribution of CAF subtypes, and frequently presents an “immune desert” phenotype with low levels of intratumoral immune cell infiltration (40). Furthermore, approximately 16% of NSMP cases exhibit loss of HLA class I molecule expression, which is significantly associated with immune evasion and poor prognosis (101) Why do patients with the same molecular subtype show divergent responses to immunotherapy? Single-cell data have unveiled the existence of “intra-subtype subtypes”. Even among POLE-mutant patients, two subpopulations, namely “immune-normalized” and “immune-exhausted”, have been identified, distinguished by the ratio of Tregs to effector T cells and the balance of co-stimulatory versus co-inhibitory receptor expression (102, 103). Collectively, these findings suggest that molecular subtypes fundamentally address the question of cellular identity composition within the tumor immune microenvironment—namely, which immune cell subpopulations are enriched and in what proportions—yet fine functional differences persist within the same identity label, representing the deeper layer of heterogeneity that can only be discerned at single-cell resolution.

#### 4.3.2 Emerging role of aggrephagy in the endometrial cancer TME

A recent single-cell sequencing-based study in endometrial cancer introduced the perspective of aggrephagy, analyzing 36,227 single cells from 5 samples and subclassifying fibroblasts, macrophages, CD8^+^ T cells, and lymphatic endothelial cells into 2 to 5 functional subsets based on the expression patterns of 44 aggrephagy-related genes. Aggrephagy is now described as a selective form of autophagy that targets misfolded or aggregated proteins for lysosomal degradation, representing a critical component of the cellular proteostasis network (104). Yuan et al. applied this concept to endometrial cancer by subclassifying TME cell populations based on the expression of aggrephagy-related genes, thereby linking a fundamental cellular homeostatic pathway to tumor microenvironment heterogeneity and clinical outcomes (105). CellChat analysis revealed extensive ligand-receptor interactions between these subsets and tumor epithelial cells, with macrophage communication being particularly active. The TUBA1A^+^ macrophage subtype was significantly associated with fewer lymph node metastases and longer survival, with immunohistochemical validation consistent with bioinformatic findings. These aggrephagy-related TME cell subsets demonstrated significant predictive value for both survival and response to immune checkpoint blockade therapy in endometrial cancer patients (105). Importantly, however, this study did not directly establish a causal or functional link between the TUBA1A+ macrophage phenotype and aggrephagy. Collectively, these findings indicate that aggrephagy-related gene signatures can inform TME cell subtyping and are associated with clinical outcomes in endometrial cancer, but whether aggrephagy itself functionally contributes to these phenotypes warrants further experimental validation.

#### 4.3.3 Three-dimensional dissection of the “subtype-driven” microenvironment in endometrial cancer

Synthesizing the above findings, the essence of the “subtype-driven” immune barrier in endometrial cancer can be understood within a three-dimensional framework: the TCGA molecular subtypes determine, at the identity dimension, the fundamental composition and abundance of tumor-infiltrating immune cells—the POLE and MSI subtypes are characterized by CD8^+^ T cell enrichment, the p53abn subtype is dominated by immunosuppression, and the NSMP subtype presents an immune desert phenotype driven by CAF heterogeneity and HLA-I loss. However, fine functional differences persist within the same molecular subtype, as exemplified by the “immune-normalized” and “immune-exhausted” subpopulations within the POLE subtype, which explains the variable therapeutic responses observed among patients with the same subtype. Furthermore, the discovery of aggrephagy-associated cell subsets suggests that functional heterogeneity at the state dimension may partially originate from differences in intracellular homeostatic regulatory pathways such as aggrephagy/autophagy. Collectively, these three dimensions indicate that immunotherapeutic strategies for endometrial cancer should precisely screen beneficiary populations based on molecular subtypes, with priority given to ICB in POLE/MSI subtypes, while simultaneously paying attention to functional heterogeneity within the same subtype and exploring individualized regimens combining targeted therapies against pathways such as aggrephagy. To systematically present the differences in immune characteristics among the three major gynecological cancers within the three-dimensional framework, we have summarized **Table 1** and **Figure 2**.

**Table 1:** Single-cell analysis of immune phenotypes and three-dimensional spatial characteristics in three gynecological cancers.

Comparison dimension	Ovarian cancer (HGSOC) (6)	Cervical cancer (9)	Endometrial cancer (10)
Predominant immune phenotype	Predominant immune phenotype: Immune-excluded^*^	Functionally disabled (TILs “present but inert”)	Subtype-dependent
Phenotypic characteristics	T cells are present in the stromal region but fail to penetrate the tumor parenchyma	Presence of immunosuppressive signals such as TGF-β, with T cell effector functions constrained by the microenvironmental signaling network	POLE ultramutated subtype characterized by high tumor mutational burden and dense immune infiltration, whereas p53-abn subtype characterized by low immune infiltration
Core obstacle in the spatial dimension	CAF/TGF-β construct physical barriers, excluding T cells from the tumor core	Spatial interactions exist but are overridden by immunosuppressive signals	Spatial distribution varies by subtype (T cells widespread in POLE; sparse in p53)
Core obstacle in the state dimension	CD8^+^ T cell exhaustion; TAMs skewed toward pro-angiogenic phenotype (Angio-Mac)	TILs are abundant but functionally constrained; HPV drives suppressive signals such as TGF-β, impairing T cell function	T cell activation in POLE and MSI; immunosuppression predominates in p53 (97, 98)
Core obstacle in the identity dimension	CAF heterogeneity (myCAF physical barrier + apCAF immunomodulation) (91, 92)	HPV^+^ enriched in CD4^+^ T cells/cDC2s; HPV⁻ enriched in CD8^+^ T cells (36)	High TMB in POLE drives neoantigen generation (38, 39); HLA-I loss in NSMP subtype (101)
Implications for ICB combination strategies	Anti-CAF/anti-TGF-β + ICB (relieve spatial barrier first)	Anti-TGF-β/anti-CD47 + ICB (relieve functional suppression)	Subtype-guided selection (POLE/MSI prioritized; p53 requires combination with other strategies)

*The immune-excluded phenotype is particularly characteristic of HRD-positive tumors with stromal restriction, which exhibit T cell infiltration but impaired intratumoral penetration.

**Figure 2:**
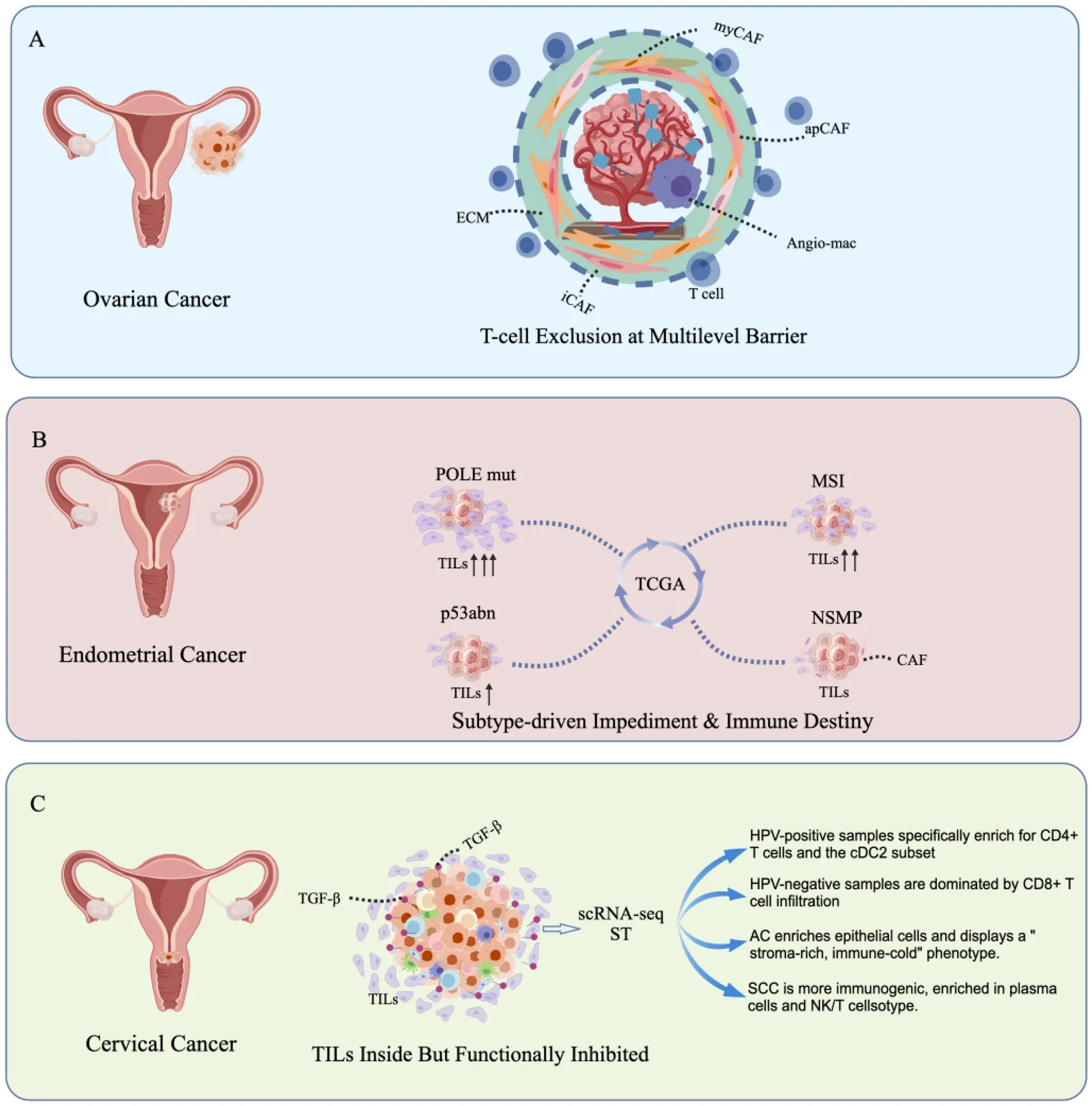
Schematic of three immune microenvironment dysfunction paradigms in major gynecological cancers. **(A)** Ovarian cancer – spatial exclusion type: Cancer-associated fibroblasts (CAFs) remodel the extracellular matrix to form a physical barrier at the tumor margin, excluding T cells from the tumor core, while pro-angiogenic macrophage subsets (e.g., Angio-Mac) further reinforce immunosuppression. **(B)** Endometrial cancer – subtype-driven type: TCGA molecular subtypes determine the baseline immune landscape. The POLE ultramutated subtype displays dense TIL infiltration (a “hot” phenotype), while the MSI subtype shows intermediate infiltration with mixed activation/suppression signals. Over 50% of p53abn cases are TIL-enriched, yet the overall microenvironment remains predominantly immunosuppressive; The NSMP subtype, featuring CAF heterogeneity and HLA-I loss, presents an ‘immune desert’ with sparse immune infiltration. **(C)** Cervical cancer – functional disability type: HPV^+^ tumors are enriched in CD4^+^ T cells and cDC2s, whereas HPV⁻ tumors are dominated by CD8^+^ T cell infiltration. Squamous cell carcinoma (SCC) is more immunogenic than adenocarcinoma (AC), with spatial crosstalk between CD8^+^ T cells and epithelial subtypes. Despite abundant TILs driven by persistent high-risk HPV infection, immune function is constrained by robust enrichment of immunosuppressive signals, notably TGF-β.

## 5 From single-cell atlas to clinical decision-making

Single-cell sequencing is driving the transition of tumor target discovery from population averages to single-cell precision. Conventional bulk RNA-seq only reflects population-level average expression and is insufficient to identify rare subpopulations and their specific molecular features. In contrast, single-cell technologies enable the profiling of thousands of cells at single-cell resolution, precisely identifying functional marker-positive subpopulations and significantly enhancing the resolution and specificity of target discovery. On this basis, several immunologically actionable targets with translational potential have been identified in gynecological cancers. However, immune evasion mechanisms differ across tumor types, and accordingly, the corresponding strategies to counteract them should be tailored accordingly.

In ovarian cancer, a tumor classically characterized by spatial exclusion, TREM2^+^ macrophages are enriched in ascites, and their expression is negatively correlated with CD8^+^ T cell activation, suggesting that they suppress immune responses through the TREM2 signaling axis. Myeloid-targeting monoclonal antibodies, including PY314 (anti-TREM2) and PY159 (anti-TREM1), have entered early-phase clinical testing; PY314 has completed a phase I trial in platinum-resistant ovarian cancer, demonstrating acceptable safety and stable disease as monotherapy and in combination with pembrolizumab (106, 107). Concurrently, single-cell analyses have identified a pro-angiogenic macrophage subpopulation (Angio-Mac) associated with poor prognosis, in which the NF-κB signaling pathway is highly activated, suggesting that intervening in NF-κB signaling may suppress tumor angiogenesis by remodeling the functional state of macrophages (35). The common rationale underlying these targets is to dismantle the spatial barrier constructed by immunosuppressive myeloid cells and aberrant angiogenesis within the ovarian cancer microenvironment. While preclinical studies in HGSOC models demonstrate that coordinated blockade of TGF-β and PD-L1 can provoke durable antitumor immunity (108), and TGF-β-driven stromal barriers are increasingly recognized as a proximal mechanism of ICB resistance in ovarian cancer (47), clinical strategies targeting this axis remain predominantly in early-phase testing. These include TGF-β receptor inhibitors such as galunisertib, bifunctional TGF-β/PD-L1 inhibitors such as bintrafusp alfa, and CAF-modulating agents, with no phase III data yet available (47, 109). Notably, the limited clinical success targeting CAFs in ovarian cancer has been explicitly acknowledged in the literature, with calls for well-designed studies with adequate sample sizes before clinical benefit can be expected (110). These strategies therefore remain mechanistically compelling and preclinically promising, but not yet clinically validated in ovarian cancer.

In cervical cancer, a “hot but inert” tumor characterized by functional disability, single-cell sequencing combined with the inferCNV algorithm has identified five tumor epithelial cell subtypes, among which the TSPAN1^+^ EPC subtype accounts for the highest proportion in both high-grade squamous intraepithelial lesions (HSIL) and cervical cancer, and exhibits a significantly elevated CNV score, suggesting greater malignant potential. This subtype has been proposed as a prognostic biomarker and a potential therapeutic target (111). Precision intervention targeting tumor epithelial subtypes may relieve the functional constraints imposed on T cells within the HPV-driven microenvironment. Furthermore, the strategy of anti-CD47 monoclonal antibody combined with radiotherapy has also received biological evidence at the single-cell level (112).

In endometrial cancer, a tumor in which immune fate is dictated by molecular subtypes, single-cell immunoprofiling has revealed the presence of T cell subpopulations co-expressing LAG-3 and TIM-3, which exhibit a deeply exhausted phenotype (113). Combination strategies simultaneously targeting LAG-3 and TIM-3 are currently under clinical investigation in endometrial cancer, including the POD1UM-204 trial evaluating retifanlimab (anti-PD-1) plus INCAGN02385 (anti-LAG-3) and INCAGN02390 (anti-TIM-3) in advanced/metastatic disease (NCT04463771) (114). Separately, PD-1/LAG-3 and PD-1/TIM-3 bispecific antibodies (tobemstomig and lomvastomig) have entered first-in-human testing in other solid tumors, though their evaluation in gynecological malignancies remains pending (115). In addition, integrated single-cell and spatial transcriptomic analyses have identified the TUBA1A^+^ macrophage subtype as being significantly associated with fewer lymph node metastases and longer survival, suggesting that the aggrephagy pathway may participate in the regulation of tumor progression (105). Collectively, these findings indicate that the translational priority in endometrial cancer lies in precisely screening ICB-responsive populations based on molecular subtypes and microenvironmental features, while concurrently exploring individualized strategies that combine targeting of novel pathways such as aggrephagy.

Despite the identification of numerous immunologically actionable targets with translational potential through single-cell omics—including anti-TREM2 monoclonal antibodies that deplete or modulate TREM2^+^ tumor-associated macrophages, which have demonstrated efficacy in preclinical ovarian cancer models (106) and have entered early-phase clinical testing in platinum-resistant ovarian cancer (107), and bispecific antibodies against LAG-3/TIM-3 in endometrial cancer that are currently under clinical investigation (113), only a limited number have advanced to large-scale clinical trials. The roots of this translational bottleneck can be examined from both technical and biological perspectives. At the technical level, single-cell sequencing has traditionally imposed stringent requirements on the quality of fresh tissue specimens, and conventionally obtained formalin-fixed or frozen samples were long considered unable to meet these standards. This is now an active area of methodological development: fixed-RNA and random-primer single-nucleus approaches recover cell types, pathway activities and clinically relevant cell states from FFPE and archived frozen tissue with fidelity comparable to fresh material, as shown by matched-sample benchmarking (116–118). Remaining limitations include reduced transcript capture, systematic reweighting of detection across RNA classes because nuclei rather than whole cells are profiled, and residual variability across tissue types and preservation conditions (117–119). The relatively high cost of detection limits its applicability to large-scale clinical cohorts (23, 120); and the analytical pipelines are complex and lack standardization, rendering cross-study comparisons of results difficult (121, 122). At the biological level, single-cell transcriptomics provides only a static snapshot of gene expression, which cannot fully represent the dynamic processes of protein function and cellular interactions—the discordance between transcript abundance and protein activity remains an inherent limitation that is difficult to circumvent when extrapolating clinical conclusions from single-cell transcriptomic data (123–125). Therefore, most of the aforementioned target discoveries currently remain at the stage of target identification and early validation, and their clinical translational value awaits confirmation through larger prospective cohort studies, multi-omics integrative analyses, and functional experiments.

## 6 Conclusion

Functional single-cell omics have fundamentally revolutionized our understanding of immune microenvironment heterogeneity in the three major gynecological cancers across the three dimensions of identity, state, and space, elevating traditional coarse population-level descriptions into a networked landscape that integrates molecular resolution, functional dynamics, and spatial architecture. This technological armamentarium provides precise molecular and spatial coordinates for addressing the respective spatial exclusion, functional disability, and subtype-driven barriers in these three gynecological cancers, offering a cell-level evidence-based systematic answer to the clinical dilemma of “why gynecological cancers, despite sharing the same organ system, exhibit profoundly divergent responses to immunotherapy”. However, the translational pathway from single-cell atlas to clinical decision-making remains confronted with multiple challenges, including technical hurdles, cost-effectiveness considerations, and biological complexity. Future efforts should focus on advancing large-scale prospective cohort validation, establishing standardized multi-omics analytical pipelines, and developing detection technologies compatible with routine clinical specimens. Concurrently, single-cell discoveries must be tightly integrated with functional validation and clinical trial design to ultimately translate the cognitive advantages afforded by single-cell resolution into precision therapeutic strategies that benefit patients.

## References

[1] Singh D Vignat J Lorenzoni V Eslahi M Ginsburg O Lauby-Secretan B . Global estimates of incidence and mortality of cervical cancer in 2020: A baseline analysis of the Who global cervical cancer elimination initiative. Lancet Glob Health. (2023) 11:e197–206. doi: 10.1016/S2214-109X(22)00501-0 36528031 PMC9848409

[2] Xu J Liao K Yang X Wu C Wu W . Using single-cell sequencing technology to detect circulating tumor cells in solid tumors. Mol Cancer. (2021) 20:104. doi: 10.1186/s12943-021-01392-w 34412644 PMC8375060

[3] Bray F Laversanne M Sung H Ferlay J Siegel RL Soerjomataram I . Global cancer statistics 2022: Globocan estimates of incidence and mortality worldwide for 36 cancers in 185 countries. CA: A Cancer J For Clin. (2024) 74:229–63. doi: 10.3322/caac.21834 38572751

[4] Sheikh MA Althouse AD Freese KE Soisson S Edwards RP Welburn S . USA endometrial cancer projections to 2030: Should we be concerned? Future Oncol. (2014) 10:2561–8. doi: 10.2217/fon.14.192 25531045

[5] Pujade-Lauraine E Fujiwara K Ledermann JA Oza AM Kristeleit R Ray-Coquard IL . Avelumab alone or in combination with chemotherapy versus chemotherapy alone in platinum-resistant or platinum-refractory ovarian cancer (Javelin Ovarian 200): An open-label, three-arm, randomised, phase 3 study. Lancet Oncol. (2021) 22:1034–46. doi: 10.1016/S1470-2045(21)00216-3 34143970

[6] Yan Y Lu J Luo H Wang Z Xu K Wang L . Decoding immune low-response states in ovarian cancer: Insights from single-cell and spatial transcriptomics for precision immunotherapy. Front Immunol. (2025) 16:1667464. doi: 10.3389/fimmu.2025.1667464 41050676 PMC12491270

[7] Matulonis UA Shapira R Santin A Lisyanskaya AS Pignata S Vergote I . Final results from the Keynote-100 trial of pembrolizumab in patients with advanced recurrent ovarian cancer. J Clin Oncol. (2020) 38:6005. doi: 10.1200/JCO.2020.38.15_suppl.600542790603

[8] Pawlowska A Rekowska A Kurylo W Panczyszyn A Kotarski J Wertel I . Current understanding on why ovarian cancer is resistant to immune checkpoint inhibitors. Int J Mol Sci. (2023) 24:10859. doi: 10.3390/ijms241310859 37446039 PMC10341806

[9] Gnanagurusamy J Krishnamoorthy S Muthusami S . Transforming growth factor-beta micro-environment mediated immune cell functions in cervical cancer. Int Immunopharmacol. (2024) 140:112837. doi: 10.1016/j.intimp.2024.112837 39111147

[10] Zhang Y Ju B Cheng R Ding T Wu J . Pd-L1 expression and immune infiltration across molecular subtypes of endometrial cancer: An integrative-analysis of molecular classification and immune subtypes. Hum Pathol. (2024) 154:105704. doi: 10.1016/j.humpath.2024.105704 39662783

[11] Tzec-Interian JA Gonzalez-Padilla D Gongora-Castillo EB . Bioinformatics perspectives on transcriptomics: A comprehensive review of bulk and single-cell rna sequencing analyses. Quant Biol. (2025) 13:e78. doi: 10.1002/qub2.78 41675508 PMC12806032

[12] Tang F Barbacioru C Wang Y Nordman E Lee C Xu N . Mrna-seq whole-transcriptome analysis of a single cell. Nat Methods. (2009) 6:377–82. doi: 10.1038/nmeth.1315 19349980

[13] Diop M Davidson BR Fragiadakis GK Sirota M Gaudilliere B Combes AJ . Single-cell omics technologies - Fundamentals on how to create single-cell looking glasses for reproductive health. Am J Obstet Gynecol. (2025) 232:S1–S20. doi: 10.1016/j.ajog.2024.08.041 40253074 PMC12090843

[14] Arya A Tripathi P Dubey N Aier I Kumar Varadwaj P . Navigating single-cell rna-sequencing: Protocols, tools, databases, and applications. Genomics Inform. (2025) 23:13. doi: 10.1186/s44342-025-00044-5 40382658 PMC12085826

[15] Jaitin DA Kenigsberg E Keren-Shaul H Elefant N Paul F Zaretsky I . Massively parallel single-cell rna-seq for marker-free decomposition of tissues into cell types. Science. (2014) 343:776–9. doi: 10.1126/science.1247651 24531970 PMC4412462

[16] Wagner A Regev A Yosef N . Revealing the vectors of cellular identity with single-cell genomics. Nat Biotechnol. (2016) 34:1145–60. doi: 10.1038/nbt.3711 27824854 PMC5465644

[17] Buenrostro JD Giresi PG Zaba LC Chang HY Greenleaf WJ . Transposition of native chromatin for fast and sensitive epigenomic profiling of open chromatin, DNA-binding proteins and nucleosome position. Nat Methods. (2013) 10:1213–8. doi: 10.1038/nmeth.2688 24097267 PMC3959825

[18] Baysoy A Bai Z Satija R Fan R . The technological landscape and applications of single-cell multi-omics. Nat Rev Mol Cell Biol. (2023) 24:695–713. doi: 10.1038/s41580-023-00615-w 37280296 PMC10242609

[19] Chen Y Liu Z Xu H Liu J Wang M Chi Y . Gene regulatory landscape dissected by single-cell four-omics sequencing. Nature. (2026) 653:942–51. doi: 10.1038/s41586-026-10322-z 41922764

[20] Chappell L Russell AJC Voet T . Single-cell (multi)omics technologies. Annu Rev Genomics Hum Genet. (2018) 19:15–41. doi: 10.1146/annurev-genom-091416-035324 29727584

[21] Hu W Zhang Y Mei J Fang X . Spatial transcriptomics in human biomedical research and clinical application. Curr Med. (2023) 2:6. doi: 10.1007/s44194-023-00023-430311153

[22] Method of the year 2020: Spatially resolved transcriptomics. Nat Methods. (2021) 18:1. doi: 10.1038/s41592-020-01042-x 33408396

[23] Wu X Yang X Dai Y Zhao Z Zhu J Guo H . Single-cell sequencing to multi-omics: Technologies and applications. biomark Res. (2024) 12:110. doi: 10.1186/s40364-024-00643-4 39334490 PMC11438019

[24] Zhou Y Jia E Pan M Zhao X Ge Q . Encoding method of single-cell spatial transcriptomics sequencing. Int J Biol Sci. (2020) 16:2663–74. doi: 10.7150/ijbs.43887 32792863 PMC7415427

[25] Andersson A Larsson L Stenbeck L Salmen F Ehinger A Wu SZ . Spatial deconvolution of Her2-positive breast cancer delineates tumor-associated cell type interactions. Nat Commun. (2021) 12:6012. doi: 10.1038/s41467-021-26271-2 34650042 PMC8516894

[26] Celik C Pan S Withnell E Wang Lam H Secrier M . Decrypting cancer's spatial code: From single cells to tissue niches. Mol Oncol. (2025) 19:3465–85. doi: 10.1002/1878-0261.70100 40711978 PMC12688177

[27] Shi W Zhang Z Xu X Tian Y Feng L Huang X . Single-cell and spatial transcriptomics integration: New frontiers in tumor microenvironment and cellular communication. Front Immunol. (2025) 16:1649468. doi: 10.3389/fimmu.2025.1649468 41112263 PMC12528096

[28] Zhang C Feng H Zhu J Hong W . Application of spatial and single-cell omics in tumor immunotherapy biomarkers. LabMed Discov. (2025) 2. doi: 10.1016/j.lmd.2025.10007642574925

[29] Sun J Xiao Y Xie L Qin D Zou Y Liu Y . Multi-scale transcriptomics redefining the tumor immune microenvironment. Biotech (Basel). (2026) 15:7. doi: 10.3390/biotech15010007 41562697 PMC12821520

[30] Zhang S Fu Q Yuan X Wang S Liu C Zhang C . Reshaping the colorectal cancer immune microenvironment: Insights from single-cell and spatial omics. Cancer Cell Int. (2026) 26:162. doi: 10.1186/s12935-026-04245-y 41782022 PMC13069743

[31] Le J Dian Y Zhao D Guo Z Luo Z Chen X . Single-cell multi-omics in cancer immunotherapy: From tumor heterogeneity to personalized precision treatment. Mol Cancer. (2025) 24:221. doi: 10.1186/s12943-025-02426-3 40855431 PMC12376342

[32] Ke H Li Z Li P Ye S Huang J Hu T . Dynamic heterogeneity of colorectal cancer during progression revealed clinical risk-associated cell types and regulations in single-cell resolution and spatial context. Gastroenterol Rep (Oxf). (2023) 11:goad034. doi: 10.1093/gastro/goad034 37360193 PMC10290555

[33] Izar B Stover E Tirosh I Rotem A Shah P Rodman C . Abstract AP19: Single–cell rna–sequencing of patient–derived ovarian cancer cells and patient–derived xenograft models. Clin Cancer Res. (2017) 23:AP19–AP. doi: 10.1158/1557-3265.Ovcasymp16-ap1936230740

[34] Izar B Tirosh I Stover EH Wakiro I Cuoco MS Alter I . A single-cell landscape of high-grade serous ovarian cancer. Nat Med. (2020) 26:1271–9. doi: 10.1038/s41591-020-0926-0 32572264 PMC7723336

[35] Yin S Li S Tu M Xu J . Single-cell landscape of the tumour immune microenvironment in human gynaecologic malignancies. Clin Transl Med. (2025) 15:e70538. doi: 10.1002/ctm2.70538 41275425 PMC12640613

[36] Su Q Tian X Li F Yu X Gong W Chen Y . Integrated multi-omics analysis of single-cell and spatial transcriptomics reveals distinct HPV-associated immune microenvironment features and prognostic signatures in cervical cancer. Front Immunol. (2025) 16:1612623. doi: 10.3389/fimmu.2025.1612623 41035636 PMC12481161

[37] Kogl J Pan TL Marth C Zeimet AG . The game-changing impact of POLE mutations in oncology-a review from a gynecologic oncology perspective. Front Oncol. (2024) 14:1369189. doi: 10.3389/fonc.2024.1369189 39239272 PMC11374733

[38] Wang T Yu D Wang J Zhu N Tang XB Chen X . Immune signatures of the POLE mutation in endometrial carcinomas: A systematic study based on TCGA data and clinical cohort validation. Front Oncol. (2023) 13:1250558. doi: 10.3389/fonc.2023.1250558 38023184 PMC10652564

[39] Zhao L Li X Jing Y Tang L Lin F Zhang Y . Biomarkers and immunotherapy in endometrial cancer: Mechanisms and clinical applications. Front Immunol. (2025) 16:1684549. doi: 10.3389/fimmu.2025.1684549 41181115 PMC12575317

[40] Li Y Qiu H Zhao Z Qi F Cai P . Single-cell technologies and spatial transcriptomics: Decoding immune low - response states in endometrial cancer. Front Immunol. (2025) 16:1636483. doi: 10.3389/fimmu.2025.1636483 40672944 PMC12263668

[41] Lee MYY Kaestner KH Li M . Benchmarking algorithms for joint integration of unpaired and paired single-cell rna-seq and atac-seq data. Genome Biol. (2023) 24:244. doi: 10.1186/s13059-023-03073-x 37875977 PMC10594700

[42] Riegel D Romero-Fernandez E Simon M Adenugba AR Singer K Mayr R . Integrated single-cell profiling dissects cell-state-specific enhancer landscapes of human tumor-infiltrating CD8(+) T cells. Mol Cell. (2023) 83:622–36:e10. doi: 10.1016/j.molcel.2022.12.029 36657444

[43] Stubenvoll A Schmidt M Moeller J Chango MAL Schultz C Antoniadou O . Single-cell transcriptomics and epigenomics point to CD58-CD2 interaction in controlling primary melanoma growth and immunity. Cancer Commun (Lond). (2025) 45:465–70. doi: 10.1002/cac2.12651 39812166 PMC11999891

[44] Liu DD Hu HY Li FF Hu QY Liu MW Hao YJ . Spatial transcriptomics meets diabetic kidney disease: Illuminating the path to precision medicine. World J Diabetes. (2025) 16:107663. doi: 10.4239/wjd.v16.i9.107663 40980298 PMC12444285

[45] Ke J Xu J Liu J Yang Y Guo C Xie B . High-resolution mapping of single cells in spatial context. Nat Commun. (2025) 16:6533. doi: 10.1038/s41467-025-61667-4 40664667 PMC12264276

[46] Dahlberg SK Bonet DF Franzen L Stahl PL Hoffecker IT . Hidden network preserved in slide-tags data allows reference-free spatial reconstruction. Nat Commun. (2025) 16:9652. doi: 10.1038/s41467-025-65295-w 41173855 PMC12579210

[47] He J Tao J Zhou Y Li H Feng W Xu Y . TGF-beta-driven T-cell exclusion in ovarian cancer: Single-cell and spatial transcriptomic views of immune low-response states. Front Immunol. (2025) 16:1698088. doi: 10.3389/fimmu.2025.1698088 41181126 PMC12575244

[48] Licaj M Mhaidly R Kieffer Y Croizer H Bonneau C Meng A . Residual ANTXR1+ myofibroblasts after chemotherapy inhibit anti-tumor immunity via YAP1 signaling pathway. Nat Commun. (2024) 15:1312. doi: 10.1038/s41467-024-45595-3 38346978 PMC10861537

[49] Wu Y Wei B Wei Z Wang H Liu Y Xu D . Single-cell and spatial transcriptomic profiling reveals distinct tumor microenvironment dynamics in cervical adenocarcinoma and squamous cell carcinoma. Commun Biol. (2025) 9:46. doi: 10.1038/s42003-025-09310-2 41420004 PMC12789533

[50] Guo J Tang B Fu J Zhu X Xie W Wang N . High-plex spatial transcriptomic profiling reveals distinct immune components and the HLA class I/DNMT3A/CD8 modulatory axis in mismatch repair-deficient endometrial cancer. Cell Oncol. (2023) 47:573–85. doi: 10.1007/s13402-023-00885-8 37847338 PMC11090934

[51] Zhao N Rosen JM . Breast cancer heterogeneity through the lens of single-cell analysis and spatial pathologies. Semin Cancer Biol. (2022) 82:3–10. doi: 10.1016/j.semcancer.2021.07.010 34274486 PMC8761219

[52] Yaman E Heyer N de Paiva CS Stepp MA Pflugfelder SC Alam J . Mouse corneal immune cell heterogeneity revealed by single-cell rna sequencing. Invest Ophthalmol Vis Sci. (2024) 65:29. doi: 10.1167/iovs.65.12.29 39432400 PMC11500044

[53] Liu T Ding Q Gou J Lu C Lu X Chen J . Single-cell and spatial transcriptomics integration reveals fam49b promotes tumor-associated macrophages polarization in colorectal cancer via the mk pathway. Front Immunol. (2025) 16:1682637. doi: 10.3389/fimmu.2025.1682637 41246334 PMC12615435

[54] Spitzer MH Nolan GP . Mass cytometry: single cells, many features. Cell. (2016) 165:780–91. doi: 10.1016/j.cell.2016.04.019 27153492 PMC4860251

[55] Bennett HM Stephenson W Rose CM Darmanis S . Single-cell proteomics enabled by next-generation sequencing or mass spectrometry. Nat Methods. (2023) 20:363–74. doi: 10.1038/s41592-023-01791-5 36864196

[56] Guinto E Chopra S Kuo IC Shin SB Messing M Cheung CY . Integration of mass cytometry and single-cell rna-sequencing of cells in bronchoalveolar lavage. Biotechniques. (2025) 77:179–90. doi: 10.1080/07366205.2025.2505347 40384395

[57] Funingana I-G Bedia JS Huang Y-W Delgado Gonzalez A Donoso K Gonzalez VD . Multiparameter single-cell proteomic technologies give new insights into the biology of ovarian tumors. Semin Immunopathology. (2023) 45:43–59. doi: 10.1007/s00281-022-00979-9 36635516 PMC9974728

[58] Stur E Peng F Teng PN Bayraktar E Hu M Corvigno S . The dynamic immune behavior of primary and metastatic ovarian carcinoma. NPJ Precis Oncol. (2025) 9:120. doi: 10.1038/s41698-025-00818-8 40281242 PMC12032089

[59] Regner MJ Wisniewska K Garcia-Recio S Thennavan A Mendez-Giraldez R Malladi VS . A multi-omic single-cell landscape of human gynecologic malignancies. Mol Cell. (2021) 81:4924–4941.e10. doi: 10.1016/j.molcel.2021.10.013 34739872 PMC8642316

[60] Mai Q Chi C Wang X Chen Y Zou Q Chen Q . Multi-omics profiling reveals sphingolipid metabolism reprogramming of tumor-conditioned mdscs in cervical cancer. BMC Cancer. (2026) 26:1122. doi: 10.1186/s12885-026-16914-2 42760508 PMC13589348

[61] Tamaki R Kogai H Sagane K Aoshima K Li SD . Clinicogenomic landscape of histologic subtypes in ovarian cancer: real-world evidence from a japanese nationwide cohort. JCO Precis Oncol. (2026) 10:e2501149. doi: 10.1200/PO-25-01149 42284540 PMC13268116

[62] Arnaudeau C Lundin C Helleday T . DNA double-strand breaks associated with replication forks are predominantly repaired by homologous recombination involving an exchange mechanism in mammalian cells. J Mol Biol. (2001) 307:1235–45. doi: 10.1006/jmbi.2001.4564 11292338

[63] Vilenchik MM Knudson AG . Endogenous DNA double-strand breaks: production, fidelity of repair, and induction of cancer. Proc Natl Acad Sci USA. (2003) 100:12871–6. doi: 10.1073/pnas.2135498100 14566050 PMC240711

[64] Soslow RA Han G Park KJ Garg K Olvera N Spriggs DR . Morphologic patterns associated with brca1 and brca2 genotype in ovarian carcinoma. Mod Pathol. (2012) 25:625–36. doi: 10.1038/modpathol.2011.183 22193042

[65] D'Angelo E Espinosa I Felicioni L Buttitta F Prat J . Ovarian high-grade serous carcinoma with transitional-like (set) morphology: a homologous recombination-deficient tumor. Hum Pathol. (2023) 141:15–21. doi: 10.1016/j.humpath.2023.08.010 37673346

[66] Rooney MS Shukla SA Wu CJ Getz G Hacohen N . Molecular and genetic properties of tumors associated with local immune cytolytic activity. Cell. (2015) 160:48–61. doi: 10.1016/j.cell.2014.12.033 25594174 PMC4856474

[67] Park J Kim JC Lee YJ Kim S Seo MK Kim SW . Unique immune characteristics and differential anti-pd-1-mediated reinvigoration potential of cd8(+) tils based on brca1/2 mutation status in epithelial ovarian cancers. J Immunother Cancer. (2024) 12:e009058. doi: 10.1136/jitc-2024-009058 38964784 PMC11227838

[68] Zou S Zhang S . Unraveling tumor associated macrophage biology in gynecological cancers for targeted therapy in the single cell omics era. iScience. (2025) 28:113924. doi: 10.1016/j.isci.2025.113924 41341850 PMC12670596

[69] Ghisoni E Benedetti F Minasyan A Desbuisson M Cunnea P Grimm AJ . Myeloid cell networks govern re-establishment of original immune landscapes in recurrent ovarian cancer. Cancer Cell. (2025) 43:1568–1586 e10. doi: 10.1016/j.ccell.2025.07.005 40749672 PMC12933722

[70] Prat J . Staging classification for cancer of the ovary, fallopian tube, and peritoneum. Int J Gynecology Obstetrics. (2013) 124:1–5. doi: 10.1016/j.ijgo.2013.10.001 24219974

[71] Patch A-M Christie EL Etemadmoghadam D Garsed DW George J Fereday S . Whole–genome characterization of chemoresistant ovarian cancer. Nature. (2015) 521:489–94. doi: 10.1038/nature14410 26017449

[72] Kurman RJ Shih I-M . The dualistic model of ovarian carcinogenesis. Am J Pathol. (2016) 186:733–47. doi: 10.1016/j.ajpath.2015.11.011 27012190 PMC5808151

[73] Matsuo K Machida H Matsuzaki S Grubbs BH Klar M Roman LD . Evolving population-based statistics for rare epithelial ovarian cancers. Gynecologic Oncol. (2020) 157:3–11. doi: 10.1016/j.ygyno.2019.11.122 31954534 PMC7526050

[74] McFarlane I Porter JM Brownsell E Ghaoui N Connolly KC Herrington CS . Ovarian carcinosarcoma is highly aggressive compared to other ovarian cancer histotypes. Front Oncol. (2024) 14. doi: 10.3389/fonc.2024.1399979 38854725 PMC11157229

[75] Integrated genomic analyses of ovarian carcinoma. Nature. (2011) 474:609–15. doi: 10.1038/nature10166 21720365 PMC3163504

[76] Zhang H Liu T Zhang Z Payne SH Zhang B McDermott JE . Integrated proteogenomic characterization of human high-grade serous ovarian cancer. Cell. (2016) 166:755–65. doi: 10.1016/j.cell.2016.05.069 27372738 PMC4967013

[77] Tu M Tang S Zhang Q Guo T Cen Y Xu X . Proteomic profiling and molecular reclassification of high-grade serous ovarian cancer identifies prognostic subtypes and immunotherapy biomarkers. eBioMedicine. (2026) 127:106248. doi: 10.1016/j.ebiom.2026.106248 42001613 PMC13101710

[78] Tan R Wen M Yang W Zhan D Zheng N Liu M . Integrated proteomics and scrna-seq analyses of ovarian cancer reveal molecular subtype-associated cell landscapes and immunotherapy targets. Br J Cancer. (2025) 132:111–25. doi: 10.1038/s41416-024-02894-2 39548315 PMC11723995

[79] Chen Y Zhu S Liu T Zhang S Lu J Fan W . Epithelial cells activate fibroblasts to promote esophageal cancer development. Cancer Cell. (2023) 41:903–918 e8. doi: 10.1016/j.ccell.2023.03.001 36963399

[80] Tsukui T Wolters PJ Sheppard D . Alveolar fibroblast lineage orchestrates lung inflammation and fibrosis. Nature. (2024) 631:627–34. doi: 10.1038/s41586-024-07660-1 38987592 PMC12088911

[81] Teng MW Ngiow SF Ribas A Smyth MJ . Classifying cancers based on t-cell infiltration and pd-l1. Cancer Res. (2015) 75:2139–45. doi: 10.1158/0008-5472.CAN-15-0255 25977340 PMC4452411

[82] Binnewies M Roberts EW Kersten K Chan V Fearon DF Merad M . Understanding the tumor immune microenvironment (time) for effective therapy. Nat Med. (2018) 24:541–50. doi: 10.1038/s41591-018-0014-x 29686425 PMC5998822

[83] Galon J Bruni D . Approaches to treat immune hot, altered and cold tumours with combination immunotherapies. Nat Rev Drug Discov. (2019) 18:197–218. doi: 10.1038/s41573-018-0007-y 30610226

[84] Lanitis E Dangaj D Irving M Coukos G . Mechanisms regulating t-cell infiltration and activity in solid tumors. Ann Oncol. (2017) 28:xii18–32. doi: 10.1093/annonc/mdx238 29045511

[85] Mlynska A Vaisnore R Rafanavicius V Jocys S Janeiko J Petrauskyte M . A gene signature for immune subtyping of desert, excluded, and inflamed ovarian tumors. Am J Reprod Immunol. (2020) 84:e13244. doi: 10.1111/aji.13244 32294293

[86] Elyada E Bolisetty M Laise P Flynn WF Courtois ET Burkhart RA . Cross-species single-cell analysis of pancreatic ductal adenocarcinoma reveals antigen-presenting cancer-associated fibroblasts. Cancer Discov. (2019) 9:1102–23. doi: 10.1158/2159-8290.CD-19-0094 31197017 PMC6727976

[87] Feng C Yang Y Li G Fang Z Yang B Zhao F . Spatial tumor evolution panorama of ovarian cancer. Cell Rep Med. (2026) 7:102666. doi: 10.1016/j.xcrm.2026.102666 41850247 PMC13006426

[88] Ohlund D Handly-Santana A Biffi G Elyada E Almeida AS Ponz-Sarvise M . Distinct populations of inflammatory fibroblasts and myofibroblasts in pancreatic cancer. J Exp Med. (2017) 214:579–96. doi: 10.1084/jem.20162024 28232471 PMC5339682

[89] Li H Courtois ET Sengupta D Tan Y Chen KH Goh JJL . Reference component analysis of single-cell transcriptomes elucidates cellular heterogeneity in human colorectal tumors. Nat Genet. (2017) 49:708–18. doi: 10.1038/ng.3818 28319088

[90] Johnson KA Lippert AEL Cornelio A Gadalla Y Shen X Kraus SG . Suppression of the myofibroblastic cancer-associated fibroblast phenotype to enhance anti-pd-1 response. J Exp Clin Cancer Res. (2026) 45:208. doi: 10.1186/s13046-026-03779-3 42432797 PMC13640252

[91] Tsoumakidou M . The advent of immune stimulating cafs in cancer. Nat Rev Cancer. (2023) 23:258–69. doi: 10.1038/s41568-023-00549-7 36807417

[92] Liu Y Chen X Dai Y Jia Y Kieffer Y Xie L . Molecular phenotypes and spatial archetypes: a new framework for cancer-associated fibroblasts. Cancer Cell. (2026) 44:1346–67. doi: 10.1016/j.ccell.2026.06.001 42349429 PMC13383123

[93] Yeh CY Aguirre K Laveroni O Kim S Wang A Liang B . Mapping spatial organization and genetic cell-state regulators to target immune evasion in ovarian cancer. Nat Immunol. (2024) 25:1943–58. doi: 10.1038/s41590-024-01943-5 39179931 PMC11436371

[94] Wang Y Tang Z Li H Zhou R Wu H Cen X . Chemoresistance evolution in ovarian cancer delineated by single-cell rna sequencing. Int J Mol Sci. (2025) 26:6760. doi: 10.3390/ijms26146760 40725006 PMC12294846

[95] Vazquez-Garcia I Uhlitz F Ceglia N Lim JLP Wu M Mohibullah N . Ovarian cancer mutational processes drive site-specific immune evasion. Nature. (2022) 612:778–86. doi: 10.1038/s41586-022-05496-1 36517593 PMC9771812

[96] Cancer Genome Atlas Research N Kandoth C Schultz N Cherniack AD Akbani R Liu Y . Integrated genomic characterization of endometrial carcinoma. Nature. (2013) 497:67–73. doi: 10.1038/nature12113 23636398 PMC3704730

[97] Mendiola M Pellinen T Ramon-Patino JL Berjon A Bruck O Heredia-Soto V . Prognostic implications of tumor-infiltrating t cells in early-stage endometrial cancer. Mod Pathol. (2022) 35:256–65. doi: 10.1038/s41379-021-00930-7 34642425

[98] Martin SD Thornton S Chow C Milne K de Barros JS Morris KA . Activated immune infiltrates expand opportunities for targeted therapy in p53-abnormal endometrial carcinoma. J Pathol. (2025) 266:292–305. doi: 10.1002/path.6429 40223796 PMC12146821

[99] Guo YE Liu Y Zhang W Luo H Shu P Chen G . The clinicopathological characteristics, prognosis and immune microenvironment mapping in msi-h/mmr-d endometrial carcinomas. Discov Oncol. (2022) 13:12. doi: 10.1007/s12672-022-00466-5 35239036 PMC8894509

[100] Marchetti M Vezzaro T Carollo M Facchetti E Masatti L Xhindoli L . The challenge of molecular stratification in no specific molecular profile endometrial cancer. Gynecol Oncol. (2025) 200:145–54. doi: 10.1016/j.ygyno.2025.07.020 40752272

[101] Grillini M Lenzi J Ceccarelli C de Biase D Maloberti T Coluccelli S . Endometrial carcinoma and immune escape: Prognostic relevance of Hla class I loss in Nsmp subtype. Histopathology. (2026) 88:442–56. doi: 10.1111/his.15531 40792549 PMC12703446

[102] Willvonseder B Stogbauer F Steiger K Jesinghaus M Kuhn PH Brambs C . The immunologic tumor microenvironment in endometrioid endometrial cancer in the morphomolecular context: Mutual correlations and prognostic impact depending on molecular alterations. Cancer Immunol Immunother. (2021) 70:1679–89. doi: 10.1007/s00262-020-02813-3 33340331 PMC8139910

[103] Brummel K Requesens M van Rooij N Workel HH Eggink FA Plat A . Spatiotemporal immune landscape and long-term immune memory in Pole-mutant endometrial cancer at the single-cell level. Cancer Immunol Res. (2025) 13:1911–24. doi: 10.1158/2326-6066.CIR-25-0083 40879333

[104] Hyttinen JMT Amadio M Viiri J Pascale A Salminen A Kaarniranta K . Clearance of misfolded and aggregated proteins by aggrephagy and implications for aggregation diseases. Ageing Res Rev. (2014) 18:16–28. doi: 10.1016/j.arr.2014.07.002 25062811

[105] Yuan Y Ren C Shu J Zhu K Li G Liu B . Single-cell sequencing reveals the role of aggrephagy-related patterns in tumor microenvironment, prognosis and immunotherapy in endometrial cancer. Front Oncol. (2025) 15. doi: 10.3389/fonc.2025.1560625 40201347 PMC11975906

[106] Binnewies M Pollack JL Rudolph J Dash S Abushawish M Lee T . Targeting Trem2 on tumor-associated macrophages enhances immunotherapy. Cell Rep. (2021) 37:109844. doi: 10.1016/j.celrep.2021.109844 34686340

[107] Yeku OO Barve M Tan WW Wang J Patnaik A LoRusso P . Myeloid targeting antibodies Py159 and Py314 for platinum-resistant ovarian cancer. J Immunother Cancer. (2025) 13:e010959. doi: 10.1136/jitc-2024-010959 40081941 PMC11907075

[108] Kment J Newsted D Young S Vermeulen MC Laight BJ Greer PA . Blockade of Tgf-B and Pd-L1 by Bintrafusp Alfa promotes survival in preclinical ovarian cancer models by promoting T effector and Nk cell responses. Br J Cancer. (2024) 130:2003–15. doi: 10.1038/s41416-024-02677-9 38622286 PMC11183086

[109] Washington C Dwivedi SKD Gunderson-Jackson C Walker J Mannel RS Holman LL . Feasibility Ib trial of Paclitaxel/Carboplatin + Galunisertib. (a small molecule inhibitor of the kinase domain of type 1 Tgf-B receptor) in patients with newly diagnosed, persistent or recurrent carcinosarcoma of the uterus or ovary. Gynecologic Oncol. (2025) 203:158–64. doi: 10.1016/j.ygyno.2025.10.021 41197229 PMC12978428

[110] Ding H Zhang J Zhang F Xu Y Yu Y Liang W . Role of cancer-associated fibroblast in the pathogenesis of ovarian cancer: Focus on the latest therapeutic approaches. Int Immunopharmacol. (2022) 110:109052. doi: 10.1016/j.intimp.2022.109052 35978506

[111] Li Y Lin Z Lin G Zhao Z Xiahou Z Cai P . Revolutionizing cervical cancer treatment: Single-cell sequencing of Tspan1+ tumor Epcs and immune checkpoints to assess drug sensitivity and optimize therapy. Front Immunol. (2025) 16:1574174. doi: 10.3389/fimmu.2025.1574174 40777026 PMC12328340

[112] Chen XJ Guo CH Wang ZC Yang Y Pan YH Liang JY . Hypoxia-induced Zeb1 promotes cervical cancer immune evasion by strengthening the Cd47-Sirpalpha axis. Cell Commun Signal. (2024) 22:15. doi: 10.1186/s12964-023-01450-4 38183060 PMC10768116

[113] Pirs B Pusic MN Roskar L Rizner TL Smrkolj S . Soluble immune checkpoints in endometrial cancer - a discovery study. Front Immunol. (2025) 16:1721822. doi: 10.3389/fimmu.2025.1721822 41357207 PMC12678237

[114] ClinicalTrials.gov . Pod1um-204: A Phase 2 Study of Retifanlimab Alone or in Combination With Other Therapies in Participants With Advanced or Metastatic Endometrial Cancer (2020). Available online at: https://clinicaltrials.gov/study/NCT04463771 (Accessed September 23, 2026).

[115] Rohrberg KS Melero I F Sanmamed M Muñoz-Couselo E Cervantes A Gambardella V . First-in-human study of Lomvastomig, a Pd-1-Tim-3 bispecific antibody, in patients with advanced and/or metastatic solid tumors. J Immunother Cancer. (2026) 14:e012729. doi: 10.1136/jitc-2025-012729 42320980 PMC13289304

[116] Liu X Naughton K Karsen SD Bentley P Duggan L Chaudhary N . Probing the feasibility of single-cell fixed Rna sequencing from Ffpe tissue. Int J Mol Sci. (2026) 27:1605. doi: 10.3390/ijms27031605 41684032 PMC12898089

[117] Chen H Chen YS Zhang S Chen J Yang H Fan T . Automated in situ microfluidic Random-Seq for robust single-nucleus and spatial total Rna profiling of diverse Ffpe specimens. Nat Commun. (2026) 17:8282. doi: 10.1038/s41467-026-74203-9 42331802 PMC13470330

[118] Yan C Corbett RD Trinh D Jin D Laskin J Bailey ML . Single-cell analysis of matched Ffpe and frozen tissue samples reveals comparable resolution of intratumoural heterogeneity. Front Genet. (2026) 17:1755978. doi: 10.3389/fgene.2026.1755978 41953136 PMC13055531

[119] Ivich A Greene CS . Integrating single-cell and single-nucleus datasets improves bulk Rna-Seq deconvolution. Cell Rep Methods. (2026) 6:101346. doi: 10.1016/j.crmeth.2026.101346 41895263 PMC13106970

[120] Qiu MZ Wang C Wu Z Zhao Q Zhao Z Huang CY . Dynamic single-cell mapping unveils Epstein–Barr virus-imprinted T-cell exhaustion and on-treatment response. Signal Transduct Target Ther. (2023) 8:370. doi: 10.1038/s41392-023-01622-1 37735150 PMC10514267

[121] Nesari AM MotieGhader H Ghorbian S . Advances and challenges in single-cell Rna sequencing data analysis: A comprehensive review. Briefings Bioinf. (2026) 27:bbaf723. doi: 10.1093/bib/bbaf723 41619215 PMC12860385

[122] Hrovatin K Moinfar AA Zappia L Parikh S Lapuerta AT Lengerich B . Integrating Single-Cell Rna-Seq Datasets with Substantial Batch Effects. BMC Genomics. (2025) 26:974. doi: 10.1186/s12864-025-12126-3 PMC12577435 41168710

[123] Peng Q Zhou P Li T . Stvcr: Spatiotemporal dynamics of single cells. Nat Methods. (2026) 23:542–53. doi: 10.1038/s41592-026-03010-3 41820580

[124] Barry CP Talbo GH Beauglehole A Ovchinnikov D Munro T Mahler S . Resolving single-cell gene expression by pseudotemporal integration of transcriptomic and proteomic datasets. Mol Cell Proteomics. (2026) 25:101475. doi: 10.1016/j.mcpro.2025.101475 41317903 PMC12892064

[125] Weinreb C Wolock S Tusi BK Socolovsky M Klein AM . Fundamental limits on dynamic inference from single-cell snapshots. Proc Natl Acad Sci USA. (2018) 115:E2467–E76. doi: 10.1073/pnas.1714723115 29463712 PMC5878004

